# Measuring Implementation Outcomes Change Over Time Using an Adapted Checklist for Assessing Readiness to Implement (CARI)

**DOI:** 10.1007/s10461-025-04614-0

**Published:** 2025-02-03

**Authors:** Beth Bourdeau, Mary A. Guzé, Greg M. Rebchook, Starley B. Shade, Demetrios Psihopaidas, Nicole S. Chavis, Janet J. Myers

**Affiliations:** 1 Center for AIDS Prevention Studies, University of California, 550 16th Street, 3rd Floor, San Francisco, CA 94143 USA; 2 Division of Policy and Data Health Resources and Services Administration, HIV/AIdS Bureau, Maryland, USA

## Abstract

In 2017, the Health Resources and Services Administration’s HIV/AIDS Bureau funded an Evaluation Center (EC) to assess the rapid implementation of 11 evidence-informed interventions at 25 HIV care and treatment providers across the U.S. The EC conducted an implementation science-based evaluation, including longitudinal assessment of implementation outcomes as defined by Implementation Outcome Framework (IOF) of the Conceptual Model for Implementation Research. The EC adapted a measure originally designed for implementation readiness to capture seven implementation outcomes and administered the measure to site leadership every six months, from intervention launch through the end of the initiative. The adapted measure demonstrated adequate internal consistency within and across time periods. Individual outcomes changed over the course of implementation, with the greatest period of growth during the first six months. Longitudinal relationships between outcomes posited to be most relevant at early, mid- or late-implementation were not evident in these analyses; rather, relationships between the outcomes were significant within time periods. Finally, there were differences in the trajectory of outcomes based on characteristics of the site’s larger context. The use of this adapted measure across multiple implementation settings, assessing multiple interventions, is an important step forward in the comparability of implementation outcomes more broadly.

## Introduction

Over the last two decades HIV public health leaders have called for greater understanding of implementation context and process in an attempt to improve the uptake of HIV interventions [1, 2, 3]. Implementation science has advanced significantly in response, promising to help HIV treatment and prevention programs expand generalizable knowledge across populations [4], support greater integration of HIV services [5], and facilitate continued uptake of many evidence-based practices [6].

The context in which organizations work has been shown to influence the success of intervention implementation across a wide range of healthcare settings [7]. *Implementation outcomes (IOs)* are a means to understand and assess the success of an intervention’s implementation [8] by examining how individuals within the organizational context perceive the practice which is being implemented. Implementation outcomes, however, can be difficult to quantify and assess longitudinally; they are more often assessed qualitatively [9] or using mixed method approaches [10]. To advance quantitative approaches to assess IOs, this paper presents the novel use of the Checklist for Assessing Organizational Readiness to Implement (CARI) Evidence-Based Practices [11] scale to assess concepts defined in the Conceptual Model for Implementation Research (aka Proctor Model) Implementation Outcomes Framework (IOF), which was administered to [25] HIV service organizations in a national federal funded initiative.

### Implementation Outcomes Framework

Implementation science has focused study on successful uptake and implementation of HIV-related programs, specifically how the context of implementation shapes both the delivery of an intervention and the mechanisms of action that influence initial, intermediate, and longer-term outcomes [12]. The most immediate of these outcomes are those indicators of implementation, i.e., implementation outcomes, or the effects of the actions taken to implement a new practice or service [13]. These outcomes are hypothesized to affect the quality of intervention delivery, which in turn influences improvements in client symptoms and functioning. For example, implementation outcomes highlight the importance of intervention acceptability with a prioritized population [14, 15] and have assisted in understanding specific implementation strategies and their downstream impact on patient outcomes in the HIV Continuum of Care [16, 17, 18].

Proctor’s IOF was developed out of a thorough review of implementation studies to determine the “necessary preconditions for attaining subsequent desired changes in clinical or service outcomes” in order to encourage consensus on the domain of possible implementation outcomes and standardize their definition [8]. The IOF is frequently used due to its comprehensive taxonomy of eight implementation outcomes: acceptability (whether implementation staff approve of the intervention), appropriateness (their perception of fit of the intervention to organizational context and client population), adoption (commitment to implementation), feasibility (perception of the ease of implementation), fidelity (implementing the intervention as intended), penetration (integrating the intervention into existing workflows), sustainability (the ability to provide the intervention long-term), and cost (the resources required to provide and maintain the intervention). Further, multiple implementation science frameworks posit that each outcome is more relevant at different stages of implementation [19, 20]. For example, acceptability and adoption are often associated with installation and the beginning phases of implementation, while feasibility and fidelity are more relevant to on-going implementation, and penetration and sustainability longer-term concerns [8, 19].

### Measuring Implementation Outcomes

Across multiple health topics, over 104 potential measures exist for the eight concepts of Proctor’s Implementation Outcome Framework [21]. A recent review of HIV prevention and treatment research found that none of the published studies explicitly identified an implementation science framework or model, typically assessed no more than four of a possible eight implementation outcomes, and all measured outcomes differently [22]. The lack of standardized approaches to measuring implementation outcomes [23] is not unique to the application of implementation science to HIV prevention and treatment. Many efforts to assess implementation use qualitative methods, such as utilizing the compilation of implementation strategies developed by Powell and colleagues [24] as codes for qualitative interviews with implementation staff [25, 26] or involving more complex multimethod approaches such as interviews, documentation review, and cost [27]. Overall, many of the approaches have been highly tailored to the implementation context and circumstances, and while notable in their ability to describe specifics, they lack the ability to be compared to other studies or other interventions.

There have been recent efforts to quantify the IOF, though available measures are limited, either due to assessing only a few implementation outcomes or due to a lack of transferability from one type of setting/intervention to any others [23, 28, 29, 30, 31]. There is still a need for consistent approaches to measuring implementation outcomes that are both pragmatic, (i.e., acceptable, compatible, easy, and useful [32, 33]) and psychometrically sound [34] thereby permitting longitudinal assessment [35, 36]. The current study, an evaluation of multiple interventions in multiple settings conducted multiple years, provided an opportunity to test an adapted measure to assess implementation outcomes that can be used in a variety of organizational contexts and at various stages of implementation.

### Current Study

From 2017 to 2021, the Department of Health and Human Services Health Resources and Services Administration (HRSA), HIV/AIDS Bureau (HAB)’s Ryan White HIV/AIDS Program (RWHAP) Part F– Special Projects of National Significance (SPNS) program conducted the *Using Evidence-Informed Interventions to Improve Health Outcomes among People Living with HIV (E2i) Initiative* to support implementation of evidence-informed interventions that aim to reduce HIV-related health disparities and improve health outcomes. E2i assessed the rapid selection and implementation of 11 interventions at 25 organizations in four focus areas [37]: programs for (1) transgender women [38] or (2) Black men who have sex with men [39]; and integration of either (3) behavioral health [40] or (4) addressing trauma [41], with HIV medical services. The Fenway Institute, in partnership with AIDS United, served as the initiative’s Coordinating Center for Technical Assistance (CCTA) to conduct the process for selection of the final interventions for each focus area, as well as to disburse funding and provide technical support [42] to each organization implementing an intervention [37].

The Center for AIDS Prevention Studies at the University of California, San Francisco (UCSF) conducted the multi-site, multi-intervention comprehensive evaluation of the initiative. The evaluation was based on the Proctor Model [8, 43] and was designed as a Hybrid Type III implementation study [44] using a Triangulation Convergence Mixed Method Model to assess the processes and outcomes of the implementation of all interventions [45]. Given the diversity of the focus areas and variation in interventions, measures and assessments had to be standardized for use initiative-wide and capable of capturing changes over time. This paper describes the selection and use of a quantitative measure for assessing longitudinal relationships among and between implementation outcomes throughout intervention implementation, specifically:

1. The measures’ internal consistency within and across multiple time points;
2. Changes in subscale scores throughout implementation;
3. Consideration of the relationships between outcome measures theorized to occur in the earlier stages of implementation to those more relevant to the later stages; and.
4. Differences by organizational context.

## Methods

The IOF includes eight concepts, one of which (i.e., cost) could be measured through a detailed, standardized, longitudinal cost assessment already included in the evaluation approach [46]. Thus, the researchers at the Evaluation Center (EC) then conducted a search for measures that (1) assessed the remaining seven concepts of the IOF; (2) were broad enough to be inclusive of all focus areas and interventions; and (3) taken together would not present a data burden for site staff to complete.

At the time of the review, we searched published literature to determine if any measures of organizational implementation settings or capacity met all three criteria. Sixteen organizational assessment measures were found as potential candidates, nine of which most closely approached assessing the concepts of interest, including the Organizational Readiness to Change Assessment (ORCA) [47], Building Blocks of Primary Care Assessment (BBPCA) [48], and the Feasibility, Evaluation Ability, and Sustainability Assessment (FEASA) [49], among others. Three lead EC researchers with expertise in implementation science (JMM) [50, 51, 52, 53], measurement of organizational variables (SBS) [52, 54, 55, 56, 57], and psychometrics (BB) [58, 59, 60] mapped all possible items from potential measures to IOF concepts, assessing them for face validity relative to #1, while also considering their ability to be used with multiple interventions (#2) and the brevity of the measure (#3). We chose to use items from the Checklist for Assessing Organizational Readiness to Implement (CARI) Evidence-Based Practices [11]. Its original form is composed of eight subscales with 5 items each: system capacity, organizational capacity, functional considerations, organizational culture/climate, senior leadership, staff capacity, implementation plan, and training. While framed as an assessment of readiness, the present tense nature of the individual items permitted a current appraisal of the organization’s relationship to the intervention being implemented. The CARI was selected as the most promising candidate, as it contained items that mapped to all IOF concepts, is applicable to any evidence-based practice or intervention (language is general to interventions broadly), and in its original form, not an undue burden in length.

### Procedures

As part of the larger evaluation, project management staff at each site completed the modified CARI in an online survey among other measures of organizational characteristics, such as type of facility, number of clients served, staffing, and workflows related to the intervention. These surveys took place first during the pre-implementation period (May 2018) and every 6 months thereafter through E2i’s completion 30 months later (December 2020).

### Participants

Using a successful approach from our other HRSA HAB evaluations [61], the Organizational Assessment was completed by a single member of the site’s leadership team (e.g., program director, Principal Investigator, supervisor). The survey as a whole was intended to assess multiple constructs of the larger Proctor framework, including site characteristics, strategies, in addition to implementation outcomes. Respondents were instructed to consult with other members of their site team to gather information and answer all questions in the survey.

### Measures

Given that the CARI subscales are organized around actors (system, organization, leadership, etc.), mapping IOF concepts often drew from multiple subscales. Three members of the research team mentioned above reviewed the items, compared them with IOF definitions, and came to consensus on which CARI items would map to each Implementation Outcome (IO). The final modified CARI had seven subscales and was composed of 28 items: Acceptance (6 items), Appropriateness (1 item), Adoption (4 items), Feasibility (7 items), Fidelity (2 items), Penetration (6 items), and Sustainability (1 item). As noted above, the items are all worded in the present tense, which permits the respondent to assess the current state within the organization. For example, the item “There is leadership support from top management in the form of a designated person responsible for implementation”—while an indicator for initial readiness—can also reflect a change in Acceptability of the intervention, should the designated position become or remain unfilled. No wording of any item was modified, and the original response options were retained: *Not even close* (1) to *We’re there* (4). Prior to analysis, all subscale and overall modified CARI scores were re-scaled from 4 points to 10 points for ease of interpretation.

### Analysis

We assessed site characteristics at baseline with descriptive statistics, utilizing proportions for organization type, and medians for clients served annually (overall and people with HIV [PWH]) and staff (overall and serving PWH). We also generated the mean proportion of PWH served among overall clients served at baseline.

We first conducted an analysis for internal consistency within and across timepoints, for both the overall modified CARI and each of the implementation outcome subscales, to ensure that all proposed subscales were functioning as coherent concepts. The overall modified CARI score is an average of all questions asked for each timepoint. The subscale score is an average of the individual questions within each subscale. Assessment of internal consistency of the overall CARI and the individual subscales was conducted using Cronbach’s alphas.

Analysis of changes in subscale scores and overall modified CARI scores over time were conducted with Generalized Estimating Equation (GEE) models, clustering by site to account for site-level differences across the initiative. The GEE model results are presented as estimated mean CARI scores and sub-scale scores for time points of interest and their 95% confidence intervals. These estimated means were also used to generate the figures. P-values presented with the estimated means indicate whether the estimated score is significant different from the baseline score, or the reference group used for the analysis.

Using this modeling technique, we compared changes in scores at each timepoint to baseline and used contrast estimates to examine differences between individual timepoints (i.e., 6-mos to 12-mos, 12-mos to 18-mos, etc.). GEE models clustered by site were also used to assess whether early IOs (acceptability, appropriateness, and adoption) predict mid-/late IOs (feasibility, fidelity, penetration, and sustainability), specifically, whether baseline scores of the early IOs predicted increased scores for the mid-/late IOs from 6-mos through 30-mos. We also assessed changes in CARI scores by organization size using GEE models. To include organization size as a predictor in the model, we applied a log_10_ transformation to the baseline annual number of all clients served. The significance level for all model estimate results was determined using α < 0.05. Study data were collected and managed using Research Electronic Data Capture (REDCap) tools hosted UCSF. REDCap is a secure, web-based software platform designed to support data capture for research studies, providing (1) an intuitive interface for validated data capture; (2) audit trails for tracking data manipulation and export procedures; (3) automated export procedures for seamless data downloads to common statistical packages; and (4) procedures for data integration and interoperability with external sources. Data were exported to SAS [62] for management and analysis.

## Results

Sites fell into one of three categories of organization type: 40% (*n* = 10) were community-based organizations, 25% (*n* = 7) were publicly funded medical clinics such as federally qualified health centers (FQHCs), and 32% (*n* = 8) were located within larger health or university systems. We observed variation in the total number of clients and the number of PWH served annually within participating sites (see Table 1). The median number of clients served was 1,875 (IQR: 905–5,203) and the median number of PWH served was 799 (292–1,475). On average, PWH represented 52% (SD: 40%, range: <1 − 100%) of all clients served. Staffing for sites varied as well. The median number of total employees was 40 (IQR: 25–125) and the median number of employees serving PWH was 29 (IQR: 20–55). On average, 70% (SD: 32%, range: <1 − 100%) of staff in participating sites serve PWH.

**Table 1:** Site characteristics at baseline

Characteristics	*N* = 25 (%)
Site Type	
Hospital/Academic Clinic	8 (32)
Publicly funded community clinic	7 (28)
Community organization	10 (40)
Median number clients served last 12 mos. (IQR)	1875 (950–5023)
Median number PWH served last 12 mos. (IQR)	799 (292–1475)
Median total number employees (IQR)	40 (25–125)
Median number employees providing HIV services (IQR)	29 (20–55)

**Fig. 1:**
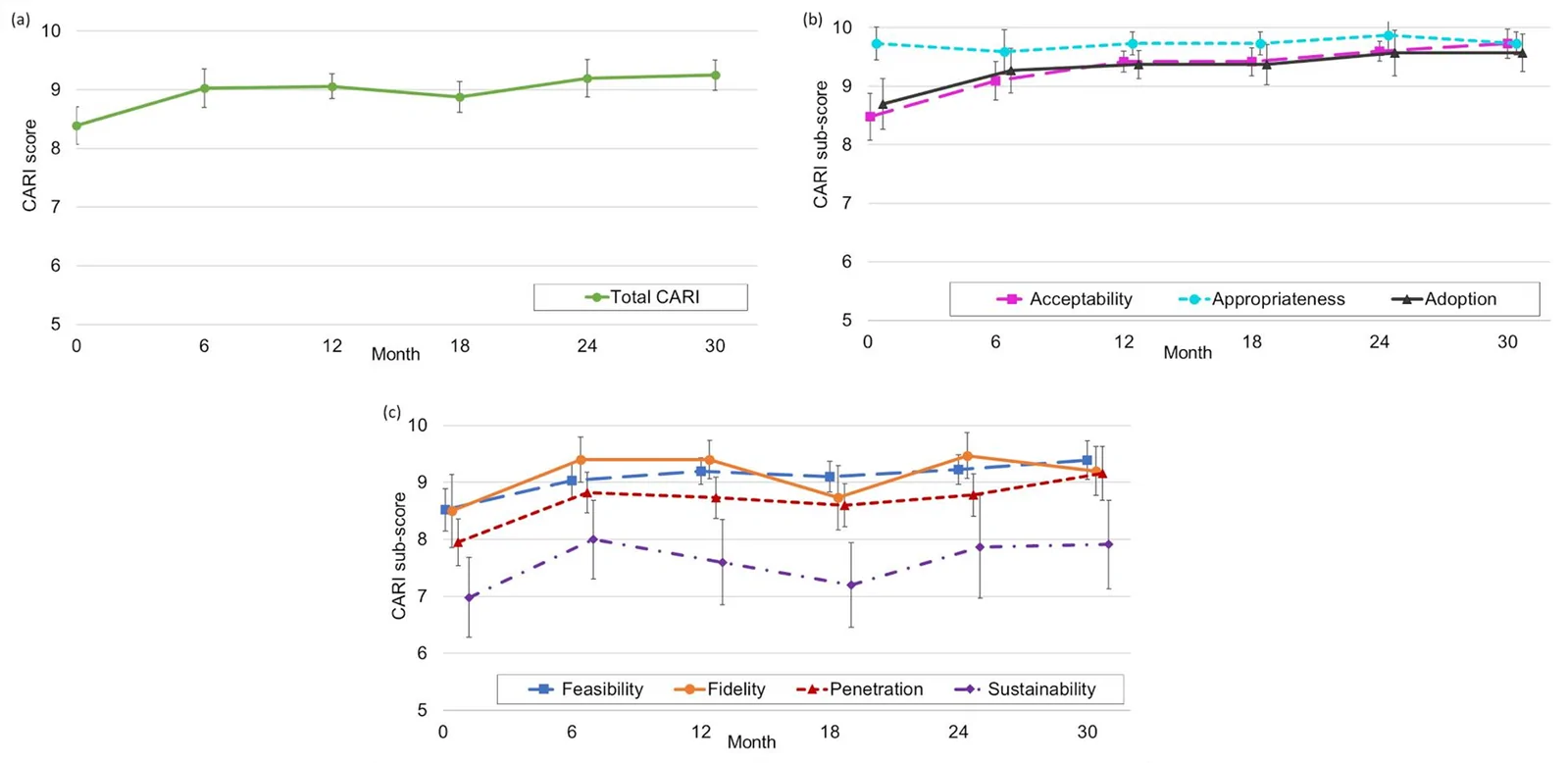
Changes in Checklist to Assess Readiness for Implementation (CARI) overall and sub-scale scores over initiative evaluation period (baseline to 30 months)

Table 2 includes a summary of the internal consistency of the CARI subscales created to reflect Proctor Model implementation outcomes. Most subscales performed well (αs = 0.79-0.92 for subscales other than fidelity). The two-item subscale which measured fidelity performed less well (α = 0.62). We employed GEE to estimate mean CARI scores (overall and for each subscale) and their 95% CIs over time. The results of these analyses are presented in Fig. 1. Overall, modified CARI scores at baseline were high (8.3; 95% CI: 8.1, 8.7) (Fig. 1A). Assessing changes in the overall modified CARI over time, we see significant increases at each timepoint compared to baseline, with the highest estimated score at 30 months of 9.2 (95% CI 9.0, 9.5; *p* <.001).

**Table 2:** Proctor implementation outcomes as applied to the checklist assessing readiness for implementation (CARI) scale questions and Cronbach’s alphas

Implementation Outcome (# questions)	Scale questions	Standardized Cronbach’s alpha^1^
Early Implementation Outcomes		
Appropriateness (1)	Senior leadership considers that the Evidence Informed Practice selected for implementation addresses an important problem / issue / gap in service delivery.	n/a
Acceptability (6)	A. All stakeholders having a role to play have been consulted about their views on the Evidence Informed Practice implementation. B. There is leadership support from top management in the form of a designated person responsible for implementation. C. This organization’s mission reflects a commitment to being a learning organization and is supportive of Evidence Informed Practice. D. Generally speaking, staff understand the mission and goals of this organization relate to Evidence Informed Practice. E. Generally speaking, staff in this organization understand what Evidence Informed Practices are and hold positive attitudes toward their use. F. Implementing the selected Evidence Informed Practice is aligned with organizational, regional, or system goals.	0.79
Adoption (4)	A. There are financial (e.g., cost effective) and/or mental health (e.g., better outcomes, meeting client needs) and/or safety reasons to implement this Evidence Informed Practice. B. Senior leadership is convinced of the value of this Evidence Informed Practice. C. Senior leadership is willing and able to lead and shape the implementation. D. Staff demonstrate readiness for practice change, and a commitment to use the new Evidence Informed Practice.	0.79
**Mid/Late Implementation Outcomes**		
Feasibility (8)	A. Technical assistance (e.g., Evidence Informed Practice training, coaching, ongoing support) is available for the Evidence Informed Practice(s) being implemented. B. Change at this time is appropriate and feasible in the life of the organization. Consider competing priorities and their timelines (e.g., accreditation demands) C. There are the physical spaces required to practice the Evidence Informed Practice selected (e.g., space for group session) D. Staff in this organization are given high levels of autonomy in their work and encouraged to ask questions. E. There are open lines of communication in place in this organization. F. There are open lines of communication in place in this organization. G. Innovation is rewarded. H. There is a rationale for the selection of staff that will be trained on the Evidence Informed Practice.	0.81
Fidelity (2)	A. There is a comprehensive implementation team in place (e.g., representatives from multiple areas of the organization). B. There is intent to monitor fidelity of the Evidence Informed Practice.	0.62
Penetration (6)	A. Human resources are adequate and available to introduce and sustain the Evidence Informed Practice. *This means:* *You intend to select only Evidence Informed Practices that match the competency levels of your staff; You are prepared to deal with changes to job requirements and staffing; You are prepared to deal with union issues; You are prepared to formally recognize the Evidence Informed Practice training / practice change accomplishments of your staff* B. There is an outcome management system in place that will enable the organization to monitor client outcomes with the new Evidence Informed Practice against preceding outcomes. C. There is a system in place to share client outcomes with staff, boards, and clients. D. There is intent to use outcome data to inform service delivery. E. The organization will consider whether policies or service/vendor contracts require revision for the implementation of the Evidence Informed Practice(s). F. There is a communications plan to share progress of the implementation plan with multiple stakeholders, regardless of their direct involvement (e.g., consider communication to your funder, board of directors, clients, community partners).	0.84
Sustainability (1)	Financial resources are adequate and available to introduce and sustain the Evidence Informed Practice. *Consider the costs of ongoing training (new staff)*, *sustainability / re-invention*, *and monitoring of fidelity to the Evidence Informed Practice*, *in addition to initial training. Costs may also be related to policy*,* system*, *or software changes.*	n/a
All combined (28)		0.93

_1_Cronbach’s alphas generated based on all responses over time. Cronbach’s alpha’s can only be calculated for subscales that have more than one question

Focusing on the implementation outcome subscales that reflect organizational readiness prior to implementation (i.e., early implementation outcomes: appropriateness, acceptability, and adoption), the mean at baseline for each subscale was above 8.0, with appropriateness highest at 9.7 (95% CI: 9.4, 10.0), acceptability at 8.5 (95% CI: 8.1, 8.9) and adoption at 8.7 (95% CI: 8.3, 9.1). Both acceptability and adoption increased significantly at 12 months (acceptability 9.4, 95% CI: 9.2, 9.6; *p* <.001, adoption 9.4, 95% CI: 9.1, 9.6; *p* =.01) and this initial increase was maintained through 30 months (Fig. 1B). We saw no change over time for appropriateness.

**Fig. 2:**
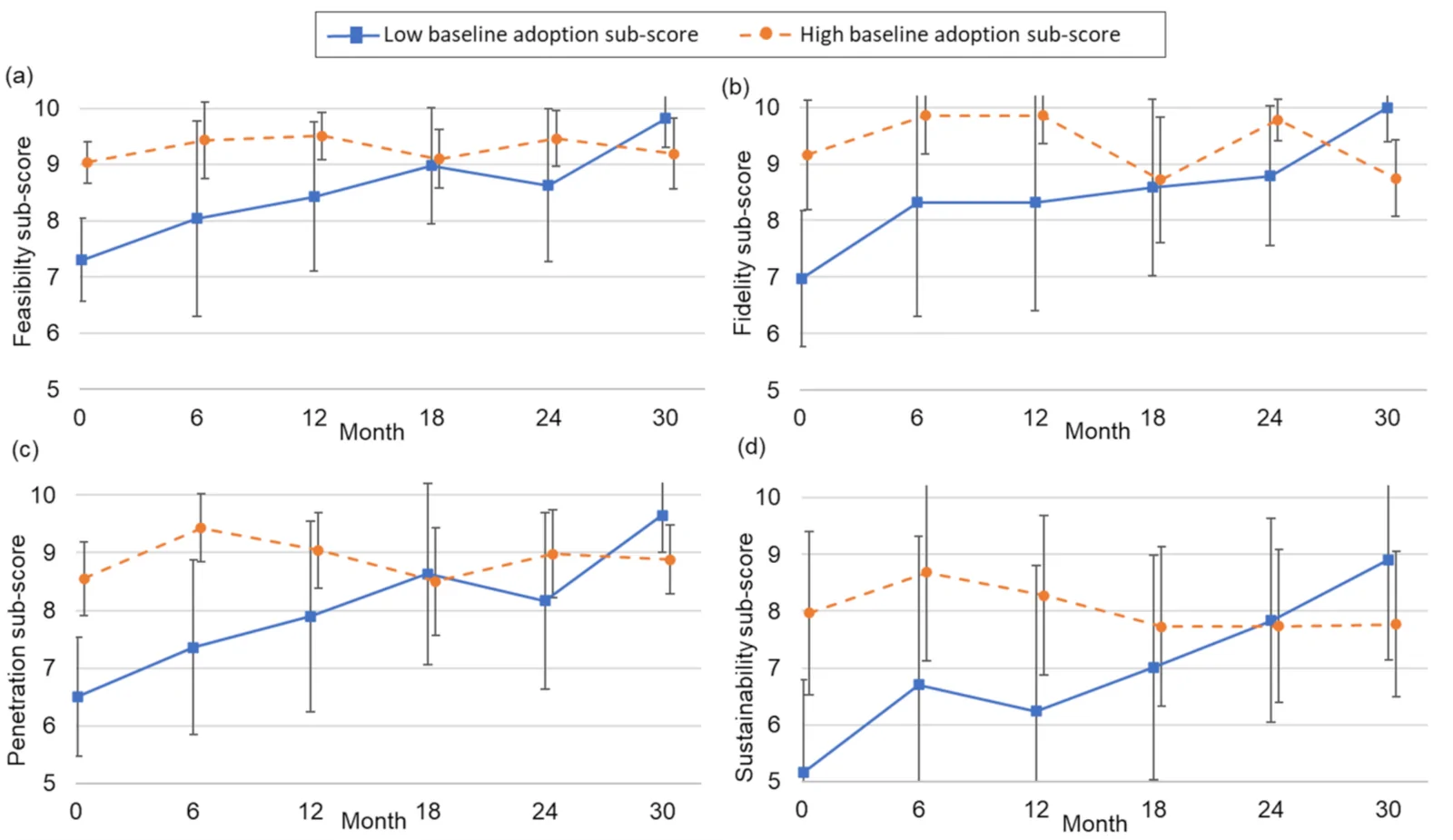
Changes in Checklist to Assess Readiness for Implementation (CARI) mid/late implementation outcome sub-scale scores (feasibility, fidelity, penetration, sustainability) by baseline adoption sub-scales scores over initiative evaluation period (baseline to 30 months)

We then examined implementation outcome subscales that reflect performance during implementation. These mid- to late implementation outcome sub-scores (feasibility, fidelity, penetration, and sustainability) ranged from a mean of 7.0 at baseline (95% CI: 6.3, 7.7) for sustainability to a mean of 8.5 at baseline (95% CI 8.1, 8.9) for feasibility. Penetration, feasibility, and fidelity all increased significantly at 6 months compared to baseline. Penetration and feasibility maintained these increases from baseline through 30 months (Fig. 2B). The fidelity sub-score dropped at 18 months but was not significantly different compared to baseline (8.7, 95% CI: 8.2, 9.3; *p* =.66). At 24 months, fidelity rebounded with a significant increase compared to baseline (9.5, 95% CI: 9.1, 9.9; *p* =.04). This difference was maintained through 30 months. Sustainability showed a significant increase only at 6 months compared to baseline (Fig. 1C).

Consistent with multiple implementation science frameworks, we also examined the relationship between early implementation outcomes and mid- to late implementation outcomes (Fig. 2). At baseline, higher early implementation outcome scores were associated with higher baseline scores for the mid-to late implementation outcomes. For example, a one-point increase in baseline acceptability was associated with a 0.41 increase in baseline feasibility (*p* =.04) and 0.56 increase in penetration (*p* <.001). However, this association does not continue over time. Higher baseline early implementation outcome scores did not predict higher mid-to late implementation outcome scores at later time points. For instance, a baseline appropriateness score of 10 was associated with a mean 6-month feasibility score of 9.15 (95% CI: 8.66, 9.63) with no significant increase seen by 30 months, 9.32 (95% CI: 8.90, 9.73). Interestingly, sites with lower baseline scores for early implementation outcomes generally showed greater increases in mid-to late implementation outcome scores over time, such that by 18 months they had closed the gap and their mid-to late implementation outcome scores did not statistically differ by baseline score for early implementation outcome. Figure 2 provides visualization of the relationship between the baseline adoption subscale score with the mid- to late implementation outcomes as an example of the associations discussed above.

**Fig. 3:**
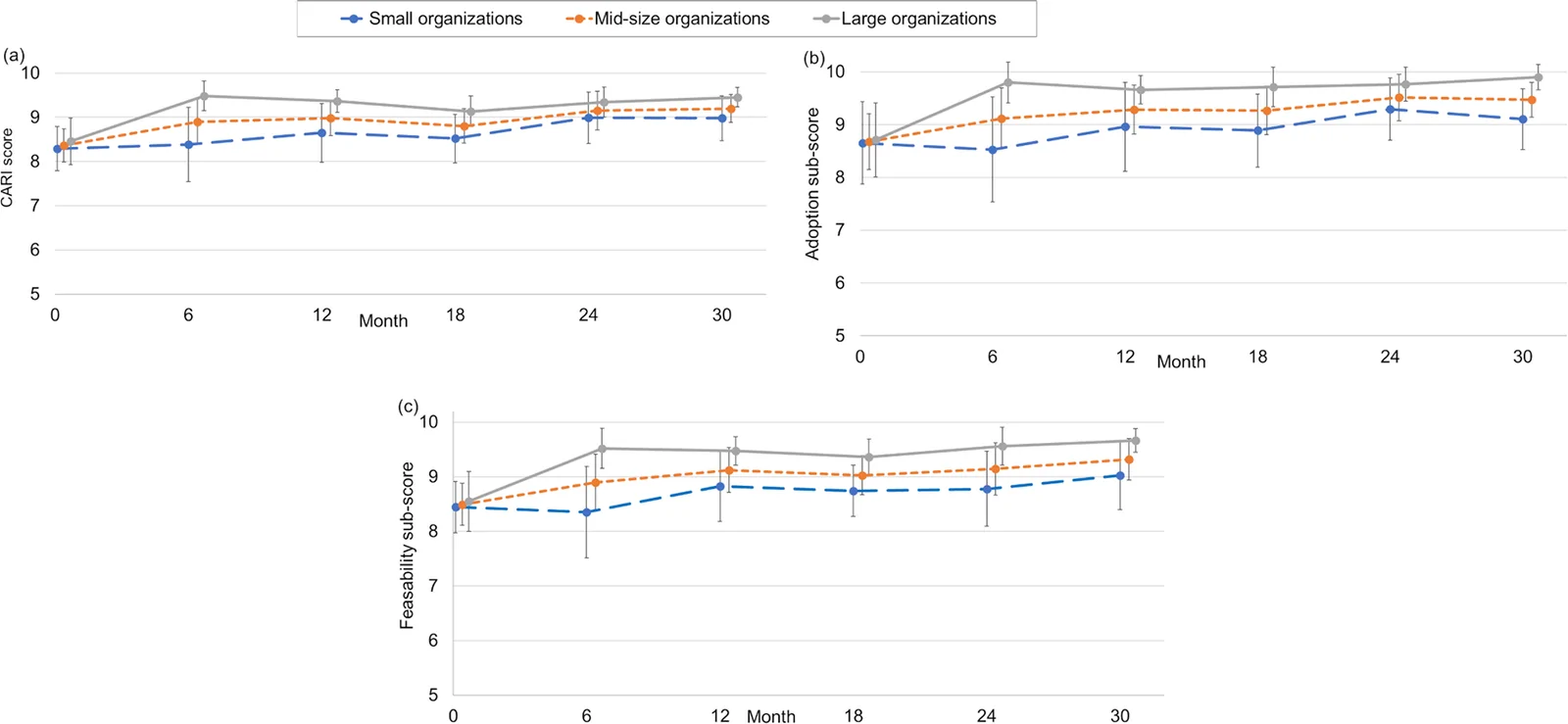
Changes in Checklist to Assess Readiness for Implementation (CARI) overall score, and adoption and feasibility sub-scale scores by organization size (annual number of clients served at baseline) over initiative evaluation period (baseline to 30 months)

Finally, we assessed characteristics of the organizations to see whether they predicted differences in implementation outcome scores over time (Fig. 3). Organization type (large healthcare setting, FQHC, community-based organization) and overall staffing (number of employees overall, employees dedicated to providing HIV care) were not significant predictors (data not shown), however, organization size (the total number of clients served annually at baseline) had some impact on implementation outcomes. At baseline there were no significant differences in implementation outcome scores by organization size; the overall CARI scores at baseline by organization size ranged from 8.23 (95% CI: 7.51, 8.94) at the smallest organization to 8.63 (95% CI: 7.45, 9.81) at the largest (*p* =.64). At 6 months, larger organization size was associated with a significantly greater increase compared to baseline in the overall CARI score, while smaller organizations saw no change at the same timepoint (*p* =.03) (Fig. 3A). This initial increase for larger organizations at 6 months was maintained (no additional increases in the overall CARI score) through 30 months (*p* =.52). Examining the individual sub-scores, we saw a similar pattern for adoption and feasibility, with a significantly greater increase compared to baseline at 6 months for larger organizations. From baseline to 6 months, each log_10_ increase in organization size was associated with a 1.02 (95% CI: 0.02, 2.02; *p* =.04) point increase in the adoption subscale score and a 0.90 (95% CI: 0.22, 1.57; *p* =.009) increase in the feasibility subscale score. The other sub-scores showed no significant difference by organization size across all time points. (Figure 3B and C).

## Discussion

We adapted a measure to advance quantitative measurement of Proctor’s IOF that assessed multiple interventions implemented in varying contexts and over the course of multiple years. Our revised CARI captured implementation outcomes across 11 different interventions delivered in 25 contexts, and both the entire scale and each subscale demonstrated stable internal consistency over time.

The measures of IOF demonstrated predictable patterns of change over the course of intervention implementation. In the initial phases of intervention installation, between baseline and 6-month administrations, sites received training from the intervention developers, hired and onboarded staff, and began the process of recruitment and enrollment of participants. During this time, all implementation outcomes saw their greatest increase and then varied little over subsequent administrations. This pattern included most of the outcomes posited as relevant for the early or mid-phases of implementation. However, the patterns in variation for fidelity and sustainability are worth additional consideration. Between the 18-month (December 2019) and 24-month (June 2020) administrations, sites saw significant shifts in their ability to deliver interventions due to the advent of COVID-related restrictions on in-person services. In light of this serious potential disruption to intervention delivery, not only did most IOs remain high, but decreases in fidelity and sustainability reversed course at subsequent timepoints, with both increasing and then staying high through the final administration. It is possible that some drift in terms of fidelity and intervention adaptations, as often occurs over the course of implementation, were reassessed in light of the service delivery shift necessitated by the pandemic response, resulting in a renewed assessment of and commitment to the core elements of the intervention. Similarly, the change to remote service delivery and expansion of telehealth or other virtual approaches perhaps also altered the perception of how sustainable the intervention was for the sites. The knowledge that the intervention might be delivered using electronic methods of connection may have benefitted the ability of sites to continue to provide the intervention services.

While the pattern of change for the implementation outcomes was different between those considered early and those considered mid- to late implementation, contrary to our expectation, we did not find evidence that the early outcomes predicted the later ones. Rather, our analyses indicated that as a group, all implementation outcomes tended to move in tandem, with the relationship *among* baseline scores stronger than those *between* baseline and later administrations. If anything, the consistency of all implementation outcomes having their greatest change happen between baseline and 6-months later highlights the importance of the work done in the installation and initial phases of implementation [8, 19].

Finally, we found that the factor making a difference in the early trajectory of implementation was the size of the organization, more than the type of organization or setting. It could be hypothesized that smaller organizations might have greater capacity for flexibility and ability to be nimble during installation and initial integration of the intervention. However, the larger organizations were the ones who saw the greatest increases in adoption and feasibility and whose scores remained the highest for the duration of the initiative. This advantage was relatively short-lived, as the smaller organizations were able to match their larger counterparts by the 18-month administration. Thus, while larger organizations may have had an initial lead via greater capacity, the smaller organizations were capable of achieving the same implementation outcomes over time, and by the end of the initiative they were indistinguishable from each other.

### Implications

This work presents a significant advancement in the development of valid, reliable measures of the Implementation Outcomes Framework, with applicability to interventions regardless of implementation setting, priority population, and intervention topic area. Further, it expands our knowledge of the relationship between implementation outcomes over time and posits a different frame for understanding their change over time, with the emphasis on the installation and early implementation phase. This period seems to be the critical time where the most growth takes place.

This work also indicates that early assessments of appropriateness, acceptability, and adoption may not be the most impactful determinants of the longer trajectory of implementation. Most of the sites studied here had high scores on all subscales from the start, a dynamic we have noticed in other studies using other individual measures of IOF concepts [63]. It is possible that there is a ceiling effect of the measure in operation. However, we posit that this is more likely due to the timing and implementation context within which studies like ours are done, i.e., after the information has been gathered by each site, stakeholders have been engaged, buy-in obtained for grant writing, and funding in hand. Ideally, measurement of IOs would begin much earlier in this process and would likely demonstrate that organizations with lower scores on IOs might be less likely to pursue or receive funding for implementation. That said, while none of the sites in this study scored exceptionally low at baseline, those that scored lower initially eventually caught up with those that started higher and had a faster rate of growth during the period between baseline and the first 6 months.

The revised CARI shows great potential for use in HIV prevention and treatment approaches, assisting in the understanding of implementation science more holistically, and utilizing implementation science as a means for improving the implementation of HIV interventions. Future directions that could enhance its utility could include linking IOs to other components of the Conceptual Model for Implementation Research, such as Implementation Strategies or intervention participant outcomes. Given the subjective nature of IOs, it will be important to link these to other quantifiable and observable data points, like provider referrals, participant recruitment, or intervention completion.

### Limitations

This study advances our knowledge of the assessment of implementation outcomes and their relationships over time; however, there are a number of issues that would urge caution in extrapolating from these results. First, while the modified CARI has face validity and good indications of internal consistency, it was originally designed to assess readiness for implementation of evidence-based practices. As this study was situated in the context of a multi-site, multi-intervention evaluation, there was not adequate time nor resources for a more systematic scale development process. Second, a more rigorous assessment would include a greater number of participating sites thus permitting opportunity for additional psychometric assessments, such as split-half reliability and confirmatory factor analyses. While the sample included in this study was diverse in terms of interventions and organization type, there were also not adequate variability in any of the other assessed organizational characteristics to permit analyses of differences with sufficient power.
